# Soaking Maize Seeds in Zeatin-Type Cytokinin Biostimulators Improves Salt Tolerance by Enhancing the Antioxidant System and Photosynthetic Efficiency

**DOI:** 10.3390/plants11081004

**Published:** 2022-04-07

**Authors:** Clara R. Azzam, Safi-naz S. Zaki, Atif A. Bamagoos, Mostafa M. Rady, Hesham F. Alharby

**Affiliations:** 1 Cell Research Department, Field Crops Research Institute, Agricultural Research Center, Giza 12619, Egypt; clara.azzam@arc.sci.eg; 2 Department of Water Relations and Field Irrigation, National Research Centre, Giza 12622, Egypt; safinsab@gmail.com; 3 Department of Biological Sciences, Faculty of Science, King Abdulaziz University, Jeddah 21589, Saudi Arabia; abamagoos@kau.edu.sa (A.A.B.); halharby@kau.edu.sa (H.F.A.); 4 Botany Department, Faculty of Agriculture, Fayoum University, Fayoum 63514, Egypt

**Keywords:** salinity, biostimulants, maize crop, growth and yield components, photosynthetic efficiency, antioxidant system, hormonal content

## Abstract

There is an urgent need for innovative strategies to raise the performance of environmentally stressed plants. The seeds of single-cross yellow *Zea mays* (L.) hybrid Giza-168 were soaked in *Cis*-(*c*-*Z*-Ck) or *trans*-zeatin-type cytokinin (*t*-*Z*-Ck) solutions at a concentration of 50 or 40 µM, respectively. Salinity stress was imposed at 0, 75 or 150 mM NaCl in the Hoagland nutrient solution (full strength) used for irrigation. The total carotenoids content was negatively affected by only 150 mM NaCl, while both 75 and 150 mM NaCl negatively affected the growth and yield components, relative water content, membrane stability index, photochemical activity, gas exchange, K^+^ and chlorophyll contents, K^+^/Na^+^ ratio, and photosynthetic efficiency. However, all of these traits were significantly improved by *c*-*Z*-Ck pretreatment and further enhanced by *t*-*Z*-Ck pretreatment compared with the corresponding controls. Furthermore, the contents of proline, soluble sugars, ascorbate, and glutathione, as well as enzymatic antioxidant activities, were significantly elevated by both salt stress concentrations and increased more by both biostimulators compared to the control. Compared to *c*-*Z*-Ck, *t*-*Z*-Ck was superior in mitigating the harmful effects of the high H_2_O_2_ levels caused by salt stress on the levels of malondialdehyde and ion leakage compared to the control. Under normal or stress conditions, *t*-*Z*-Ck pretreatment was better than *c*-*Z*-Ck pretreatment, while both positively affected maize hormonal contents. As a result, *t*-*Z*-Ck is recommended to enhance the growth and productivity of maize plants by suppressing the effects of oxidative stress caused by saline water irrigation.

## 1. Introduction

Biotic and abiotic stressors have a negative impact on a plant’s growth, and production. Abiotic stress costs USD 100 million per year due to product reduction and loss [1]. As the most critical factor, salinity limits crop productivity, and it has become a more serious issue in many parts of the world [2]. Soil salinity threatens plant cultivation worldwide. It harms growth, physiology, and metabolism in plants. Because most plants are salt-sensitive, their productivity decreases with the accumulation of salts in irrigated soil [3].

Plants’ behavioral responses to salinity are complex, and they employ a variety of mechanisms when exposed to salinity. For soils and water damaged by salinity, engineering methods have been applied to increase farm production, but it appears that achieving elevated targets using these methods is difficult [4]. High salt build-up causes “physiological drought”, which impedes penetration and reduces the water potential of the soil [5,6,7,8,9,10,11]. Salinity-exposed plants alter their metabolisms to adapt to new adverse conditions. For adaptation, plants can accumulate osmoregulatory substances such as proline and endogenous hormones to overcome the effects of salt stress. Plant tissue produces and accumulates more free amino acids, particularly proline, under stress, including salinity and drought. Proline regulates cytoplasmic osmotic potential as an osmoregulatory substance. As a result, proline may have acted as a metabolic marker in response to stress [10,12,13,14].

This adversity is worsening by the day. Thus, it must be challenged with additional research works to innovate simple techniques for crop producers (farmers) to apply to prevent the loss of agricultural production in plants under stress. Plants grown under salt stress suffer from soil erosion and Na^+^, Cl^−^, and SO_4_^−^ ions in the soil [15]. Salt stress in plants causes a variety of physio-biochemical responses, affecting most metabolic processes. Further, salinity causes water restriction even in fully irrigated soils due to reduced osmotic potential and the inhibition of gibberellic acid synthesis [16]. Salinity impairs a plant’s capacity to use water by disturbing cell turgor and negatively affecting nutrient balance, gas exchange, photosynthesis, cell expansion, and other cell metabolic processes. It also inhibits enzymatic catalysts, such as Rubisco enzymes, and increases Na^+^ and Cl^−^ ions, ultimately leading to plant death [17]. Osmolyte accumulation occurs due to salt stress, which reduces growth. This finding is due to increasing ROS, lipid peroxidation, and Na^+^ accumulation, and decreasing the contents of K^+^ and Ca^2+^ in maize [18].

The plant develops/adopts different protective antioxidants, such as superoxide dismutase, catalase, ascorbate and glutathione peroxidases, glutathione, ascorbate, proline, and others, for stress tolerance [19,20,21]. Plants, in general, are not able to withstand high stress levels due to the insufficient endogenous components of their antioxidant system. Exogenously employed antioxidants (for example, *cis*- and *trans*-zeatin-type cytokinins) should thus be advised to improve plant tolerance to salt stress [22]. Cytokinins (CKs) are phytohormones derived from adenine that regulate many aspects of plant physiology. Thus, they can improve plant tolerance to abiotic stress [23].

The application of biostimulants in agriculture to increase crop yields under stressful conditions has received attention recently [24,25,26,27]. *Cis*-zeatin-type cytokinins (*c*-*Z*-Ck) are a type of cytokinin (CK) that has received less attention than trans-zeatin (*t*-*Z*-Ck) isomers or other highly active CKs. The low activity of *c*-*Z*-Ck in traditional CK bioassays is the primary reason for its lack of interest. However, research on *c*-*Z*-Ck has been restrained by a lack of appropriate methods for determining their concentrations in plant tissues. Both *t*-*Z*-Ck and *c*-*Z*-Ck are characterized by their chromatographic behavior. Thus, the isolation and identification of *c*-*Z*-Ck are closely related to the development of new analytical methods capable of separating different zeatins. Although the reconstructions are difficult, analyses based on low-resolution chromatography methods may account for the mixture of zeatin isomers in their levels. In contrast, perhaps a few investigations have ignored them [22].

The purpose of this research was to investigate whether soaking maize seeds in two zeatin-type cytokinins (*cis*- and *trans*-) has any positive effects on growth, physiological traits (plant water status and photosynthetic efficiency), osmotic protective substances, components of the antioxidant system, plant hormones, and the ratio of K^+^/Na^+^ in maize plants exposed to stress through 75 or 150 mM NaCl.

## 2. Materials and Methods

### 2.1. Growth Conditions, Treatments, and Layout of Experiments

Giza-168, an Egyptian commercial single-cross yellow maize (*Zea mays* L.) hybrid, was kindly procured from the Agricultural Research Center. Simultaneously, three pot trials were accomplished in a greenhouse under temperatures that averaged 36 ± 4 (for the day whose mean length was 13 h) and 20 ± 3 °C (for the night whose mean length was 11 h), along with humidity levels of 61.8–66.4%.

**Figure 1:**
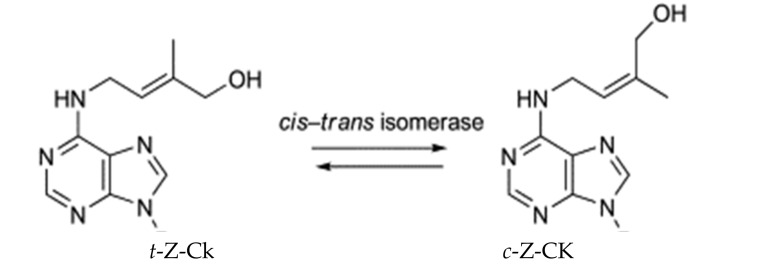
The *cis–trans* isomerization of cytokinin.

Two solutions at 50 and 40 µM were developed from *cis*- (*c*-*Z*-Ck) and *trans*-zeatin (*t*-*Z*-Ck), respectively. The *cis*–*trans* isomerization of cytokinin is shown in Figure 1. These concentrations produced the best growth of maize plants among the concentrations used, such as the 10, 20, 30, 40, 50, and so on up to 100 μM *c*-*Z*-Ck and *t*-*Z*-Ck solutions in a preliminary study (data not shown). The seeds were placed in the solutions for 24 h for soaking. This soaking period produced the best growth of maize plants among the periods used, such as 12, 18, 24, 30, and 36 h in a preliminary study (data not shown). Salinity stress was applied to plants by irrigation with a full-strength nutrient solution containing 0, 75, or 150 mM NaCl [28]. The levels 75 and 150 mM NaCl were selected for this study based on a preliminary study (data not shown), where the level 50 mM did not cause any significant damage to maize plants, while the level 150 mM NaCl was the most harmful to maize plants without death, as the plants were killed with the use of a level of more than 150 mM NaCl.

The salt-free nutrient solution was employed to irrigate the non-stressed plants, while the nutrient solutions employed for irrigation of stressed plants contained NaCl salt at 75 or 150 mM to comprise the following nine treatments: (1) Control (0 mM NaCl), (2) 50 µM *c*-*Z*-Ck, (3) 40 µM *t*-*Z*-Ck, (4) 75 mM NaCl, (5) 50 µM *c*-*Z*-Ck + 75 mM NaCl, (6) 40 µM *t*-*Z*-Ck + 75 mM NaCl, (7) 150 mM NaCl, (8) 50 µM *c*-*Z*-Ck + 150 mM NaCl, and (9) 40 µM *t*-*Z*-Ck + 150 mM NaCl.

The components of Hoagland’s nutritive solution (pH 5.9) were Ca(NO_3_)_2_ × 4 H_2_O (1250 μM), KNO_3_ (1250 μM), KH_2_PO_4_ (250 μM), MgSO_4_ × 7 H_2_O (500 μM), H_3_BO_3_ (11.6 μM), MnCl_2_ × 4 H_2_O (2.4 μM), ZnSO_4_ × 7 H_2_O (0.24 μM), CuSO_4_ × 5 H_2_O (0.08 μM), Na_2_MoO_4_ × 2 H_2_O (0.13 μM), and Fe^3+^-EDTA^+^ (22.5 μM).

As described in [29], *c*-*Z*-Ck and *t*-*Z*-Ck (Olchemim Ltd., Olomouc, Czech Republic) were procured as dilutions from stocks (100 mM) in 0.5 M NaOH (Roth, Bavaria, Germany). Two liters of *c*-*Z*-Ck and *t*-*Z*-Ck solutions were sufficient to soak 1 kg of seeds for 24 h. The same volume of distilled water was prepared to soak the control seeds for the same period of time. Under shade, the seeds were re-dried utilizing a forced-air dryer [30]. For 1 h, the seeds were sterilized with filtered calcium hypochlorite (1%) solution and then washed with sterilized-deionized water. Plastic pots with a diameter and depth of 42 and 40 cm, respectively, were used. One viable seed was planted in each pot at a 3 cm depth after being filled with 15 kg of pure, deionized sand.

All treatments were initially watered with the nutrient solution at 100% of field capacity up to 20 days after sowing (DAS). Then, saline nutrient solutions were prepared to irrigate all pots of saline treatments up to 60 DAS. All treatments were watered with the nutrient solution at 2 day intervals up to the end of the experiments (85 DAS). Inductively coupled plasma atomic emission spectrometry (ICP-AES, IRIS-Advan type, Thermo, MA, USA) was utilized to control the saline treatments.

All treatments were set as a completely randomized design (CRD). Twenty pots representing four replicates were assigned to each treatment. Sixty DAS, the samples were collected to evaluate growth parameters, physio-biochemical attributes, phytohormones, and the activities of different antioxidants. Yields were determined 85 DAS at the end of the experiments.

### 2.2. Growth, Yield Components, and Photosynthesis Efficiency Determinations

In each treatment, leaf number was recorded per each plant 60 DAS. The area of plant leaves was measured utilizing a LI-3100C Area Meter (LI-COR, Lincoln, NE, USA). Fresh roots and shoots were weighed immediately after sample collection. The samples were placed in an oven at 70 °C until their weights were stable. Then, the dried roots and shoots were weighed.

Acetone (80%) was utilized to extract chlorophylls and carotenoids from the fresh leaves to assess their contents [31]. The supernatants were measured using spectrophotometers at 663, 645, and 480 nm. According to Li et al. [32], the PAM fluorimeter (H. Walz, Effeltrich, Germany) was used to assess chlorophyll fluorescence in fresh leaf samples. At 20, 25, and 30 days after applying the stress treatments (40, 45 and 50 DAS), the gas exchange indices (stomatal conductance, transpiration rate, and net photosynthetic rate) were assessed using the infrared LCA-4 model-gas analyzer (Anal. Dev. Co., Hoddesdon, UK). PSII maximum quantum yield (Fv/Fm) was computed with the formulae of Maxwell and Johnson [33]. Photochemical activities in the fresh leaves were determined according to Jagendorf [34] and Avron [35].

### 2.3. Relative Water Content (RWC), Membrane Stability Index (MSI), Malondialdehyde (MDA), Electrolyte Leakage (EL), Soluble Sugars, Proline, Ascorbic Acid (AsA), and Glutathione (GSH) Determinations

The RWC [36], MSI, and EL [37] were evaluated in the blades of fully expanded upper leaves after excluding leaf midribs. Lipid peroxidation was determined by determining the content (µmole g^−1^ FW) of MDA. MDA was assessed using the extract as of the method for the H_2_O_2_ evaluation [38], and the contents were computed. Ethyl alcohol 96% (*v*/*v*) was utilized to extract and measure soluble sugar contents (as mg g^−1^ DW) [39]. The leaf extract (100 μL) was boiled with anthrone reagent, freshly prepared using sulfuric acid (72% *v*/*v*), for 10 min. After cooling, absorbance readings were taken at 625 nm. Free proline content (as μmol g^−1^ DW) was determined following the procedures in [40]. Fresh leafy samples were collected to evaluate AsA contents (µmol g^−1^ FW), as detailed in [41]. The same leafy samples were used to assess GSH contents (µmol g^−1^ FW), as detailed by Griffith [42].

### 2.4. Assaying Antioxidant Enzyme Activities and Hormones Assessment

A 200 mg freeze-dried leafy sample was homogenized using K-phosphate buffer (pH 7.0) to extract SOD, CAT, and GPX (GSH-peroxidase). Another buffer containing AsA (2.0 mM) and EDTA (100 µM) was used to extract APX (AsA-peroxidase). Nylon pieces were used to filter the homogenates. Centrifugation (12,000× *g*) for a quarter of an hour was also practiced. All procedures described were performed at 4 °C. The extracts were used immediately, and otherwise maintained at −25 °C. The activities of CAT (µM H_2_O_2_ min^−1^ g^−1^ protein), APX (µM H_2_O_2_ min^−1^ g^−1^ protein), GPX (µM H_2_O_2_ min^−1^ g^−1^ protein), and SOD (U mg^−1^ protein) were assayed by the application of the procedures detailed in [43,44,45,46], respectively.

The blades without midribs of fresh leafy samples were frozen in liquid N. Then, the frozen samples were ground. *Cis*- and *trans*-zeatin-type cytokinin and total cytokinins were extracted and analyzed [47].

### 2.5. Assessment of K^+^, Na^+^, and Cl^−^ Contents

The dried and powdered leafy samples were assigned to assess the K^+^, Na^+^, and Cl^−^ contents after the acidic digestion. Cl^−^ content [48], as well as K^+^ and Na^+^ contents [49], were determined following the corresponding procedures.

### 2.6. Determination of Yield and Yield Components

Eighty-five DAS, all cobs were collected from each plant to count the average number of cobs per plant. The cobs were sun-dried for ten days. Then, the grains were extracted from the cobs after shelling to calculate plant grain yield and the average weight of 100 grains.

### 2.7. Data Analysis

The ANOVA technique was applied to statistically analyze all study data [50]. Statistix^®^ analytical software, version 8.1 (Copyright 2005, Tallahassee, FL, USA) was used. The LSD technique was applied to compare treatment means at *p* ≤ 0.05, based on Tukey’s test.

## 3. Results

### 3.1. Components of Maize Growth and Yield

Seed pretreatment using 50 µM *cis*-zeatin-type cytokinin (*c*-*Z*-Ck) or 40 µM *trans*-zeatin-type cytokinin (*t*-*Z*-Ck) did not affect the weights of the fresh and dry shoots and roots, as well as the number and area of plant leaves of maize (Giza-168) compared to the control (Table 1).

Maize plants stressed with 75 mM NaCl displayed a decrease in shoot fresh weight by 24.7%, shoot dry weight by 20.8%, root fresh weight by 24.7%, root dry weight by 25.4%, plant leaf number by 26.9%, and leaf area plant^−1^ by 29.9% compared to the control. Further, exposing maize plants to 150 mM NaCl further decreased shoot fresh weight by 52.0%, shoot dry weight by 48.1%, root fresh weight by 47.1%, root dry weight by 51.2%, number of leaves plant^−1^ by 48.2%, and leaf area plant^−1^ by 53.7% compared to the control. However, pretreatment with *c*-*Z*-Ck and *t*-*Z*-Ck mitigated the 75 and 150 mM NaCl stress influences and noticeably increased all components of plant growth compared to the corresponding stressed controls (75 and 150 mM NaCl). Pretreatment with *t*-*Z*-Ck exceeded *c*-*Z*-Ck under both 75 and 150 mM NaCl concentrations and exceeded the stressed control by 10.9% and 16.3% for the shoot fresh weight, 11.6% and 10.2% for the shoot dry weight, 13.5% and 17.5% for the root fresh weight, 12.1% and 20.0% for the root dry weight, 11.1% and 13.8% for the number of leaves plant^−1^, and 20.6% and 16.0% for the leaf area plant^−1^, respectively, under the stress conditions of 75 and 150 mM NaCl (Table 1).

### 3.2. Leaf Photosynthetic Pigments and Photosynthetic Efficiency

Seed pretreatment using 50 µM *c*-*Z*-Ck or 40 µM *t*-*Z*-Ck did not affect the total chlorophylls, total carotenoids, Fv/Fm, and photochemical activity of maize (Giza-168) compared to the control (Table 2). Exposing maize plants to salt stress with 75 mM NaCl decreased the total chlorophylls, Fv/Fm, and photochemical activity by 29.4%, 16.9%, and 28.3%, respectively, compared to the control, while it increased the total carotenoids by 11.3%. In addition, compared to the control, exposing maize plants to 150 mM NaCl further decreased by 64.9%, 30.2%, 38.6%, and 45.4%, respectively. However, *c*-*Z*-Ck and *t*-*Z*-Ck pretreatment mitigated the harmful impacts of 75 and 150 mM NaCl. The *c*-*Z*-Ck and *t*-*Z*-Ck significantly increased the leaf pigment contents and photosynthesis efficiency compared to the corresponding controls. Pretreatment with *t*-*Z*-Ck exceeded *c*-*Z*-Ck under both 75 and 150 mM NaCl concentrations, exceeding the stressed control by 30.5% and 39.7% total chlorophylls, 9.3% and 23.1% for total carotenoids, 15.9% and 21.5% for Fv/Fm, and 28.5% and 31.8% for photochemical activity, respectively, under the stress conditions of 75 and 150 mM NaCl (Table 2).

### 3.3. Leaf Gas Exchange

Seed pretreatment using 50 µM *c*-*Z*-Ck or 40 µM *t*-*Z*-Ck did not affect the parameters of gas exchange (net photosynthetic rate, transpiration rate, and stomatal conductance) compared to the control (Table 3).

Exposing maize plants to 75 mM NaCl reduced the three gas exchange parameters by 31.8%, 25.6%, and 29.0%, respectively, compared to the control. Exposing maize plants to 50 mM NaCl further decreased them by 42.8%, 40.3%, and 45.2%, respectively, compared with the control. However, pretreatment with *c*-*Z*-Ck and *t*-*Z*-Ck attenuated the 75 and 150 mM NaCl influences and noticeably raised the leaf gas exchange parameters of maize compared to the corresponding stressed controls (75 and 150 mM NaCl). Pretreatment with *t*-*Z*-Ck exceeded *c*-*Z*-Ck under both 75 and 150 mM NaCl concentrations and exceeded the stressed control by 31.5% and 23.2% for net photosynthetic rate, 26.1% and 16.9% for transpiration rate, 27.9% and 24.4% for stomatal conductance, respectively (Table 3).

### 3.4. Relative Water Content (RWC), Membrane Stability Index (MSI), and Ion Leakage (EL)

Seed pretreatment using 50 µM *c*-*Z*-Ck or 40 µM *t*-*Z*-Ck did not affect the RWC, MSI, and EL of maize (Giza-168) leaves compared to the control (Table 4). Exposing maize plants to salt stress with 75 mM NaCl decreased RWC and MSI by 23.6% and 32.6%, respectively, compared with the control. Contrastingly, electrolyte leakage (EL) was increased by 37.0%. Further, exposing maize plants to salt stress with 150 mM NaCl further decreased by 46.6% and 48.5%, respectively, compared to the control, while electrolyte leakage (EL%) was increased by 51.4%. However, pretreatment with *c*-*Z*-Ck and *t*-*Z*-Ck mitigated the 75 and 150 mM NaCl stress impacts and significantly elevated the RWC and MSI of maize leaves compared to the stressed 75 and 150 mM NaCl control, and vice versa for electrolyte leakage. Pretreatment with *t*-*Z*-Ck significantly exceeded *c*-*Z*-Ck under both stress concentrations (75 and 150 mM NaCl), which exceeded the stressed control by 23.5% and 25.9% for RWC (%) and 32.9% and 27.4% for MSI (%), respectively, under the stress conditions of 75 and 150 mM NaCl, and vice versa for EL (%), as shown in Table 4.

### 3.5. Lipid Peroxidation, Osmoprotectants, and Antioxidants Contents of Maize

Seed pretreatment using 50 µM *c*-*Z*-Ck or 40 µM *t*-*Z*-Ck did not affect the malondialdehyde (MDA) level, hydrogen peroxide (H_2_O_2_) as an oxidative stress biomarker, osmoprotectant contents (proline and soluble sugars), and antioxidant contents (ASA and glutathione; GSH) compared to the control (Table 5). Exposing maize plants to salt stress with 75 mM NaCl increased the oxidative stress biomarkers by 33.3% and 39.4%, respectively, for MDA and H_2_O_2_, compared to the control. Further, exposing maize plants to salt stress with 150 mM NaCl further increased oxidative stress biomarkers by 45.2% and 62.5%, respectively, for MDA and H_2_O_2_, compared to the control. The contents of H_2_O_2_ and MDA were noticeably decreased by *c*-*Z*-Ck and *t*-*Z*-Ck compared to the stressed control. Seed treatment with *c*-*Z*-Ck awarded better findings than *t*-*Z*-Ck. In addition, *c*-*Z*-Ck pretreatment reduced MDA by 19.9% and 8.4%, respectively, under 75 and 150 mM NaCl stress conditions, while *t*-*Z*-Ck reduced MDA content by 34.2% and 15.6%, respectively, under 75 and 150 mM NaCl stress compared to the stressed control (Table 5). Pretreatment with *c*-*Z*-Ck reduced H_2_O_2_ by 17.6% and 14.1%, respectively, under 75 and 150 mM NaCl stress conditions, while *t*-*Z*-Ck reduced H_2_O_2_ content by 40.4% and 29.6%, respectively, under 75 and 150 mM NaCl stress conditions, compared to the stressed control (Table 5). However, seed treatment with *c*-*Z*-Ck and *t*-*Z*-Ck mitigated the 75 and 150 mM NaCl stress impacts and significantly elevated proline, soluble sugars, ASA, and glutathione content compared to the stressed 75 and 150 mM NaCl controls. Pretreatment with *t*-*Z*-Ck exceeded *c*-*Z*-Ck under both stress concentrations (75 and 150 mM NaCl), which exceeded the stressed control by 37.8% and 28.1% for proline, 33.3% and 32.7% for soluble sugars, 19.9% and 19.3% for AsA, and 18.1% and 20.2% for glutathione content, respectively, under the stress conditions of 75 and 150 mM NaCl. The seed treatment was more effective under stress than under normal conditions (Table 5).

### 3.6. Enzymatic Antioxidant Activities of Maize

**Figure 2:**
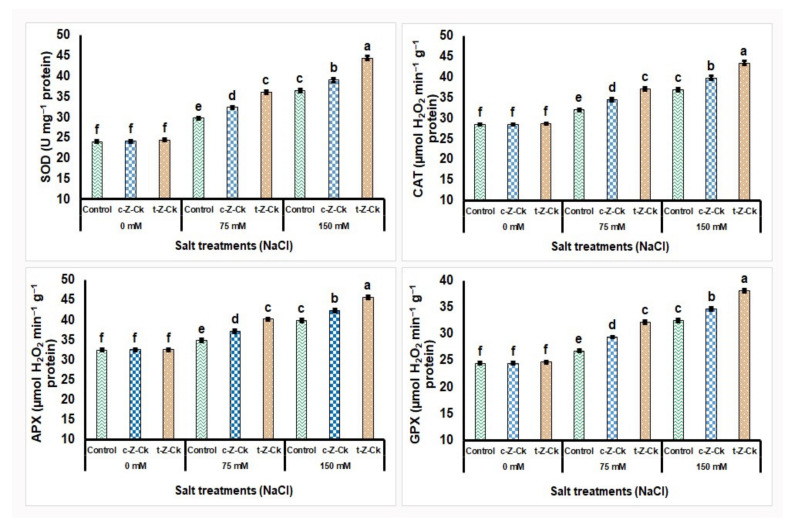
Seed soaking in 50 µM *c*-*Z*-Ck or 40 µM *t*-*Z*-Ck affected the enzymatic antioxidant activities of maize (Giza-168) plants exposed to salinity stress. Means with similar letters in each column are not significant at *p* ≤ 0.05, depending on Tukey’s test. SOD refers to superoxide dismutase, CAT refers to catalase, APOX refers to ascorbate peroxidase, and GPOX refers to glutathione peroxidase.

Seed pretreatment using 50 µM *c*-*Z*-Ck or 40 µM *t*-*Z*-Ck did not affect the enzymatic antioxidant activities of maize (Giza-168) compared to the control (Figure 2). Exposing maize plants to salt stress with 75 mM NaCl increased the enzymatic antioxidant activities by 19.1%, 10.9%, 6.9%, and 8.6%, respectively, for SOD, CAT, APX, and GPX compared to the control. Further, exposing maize plants to salt stress with 150 mM NaCl further increased the enzymatic antioxidant activities by 34.0%, 22.7%, 15.6%, and 24.6%, respectively, for SOD, CAT, APX, and GPX compared to the control. However, pretreatment with *c*-*Z*-Ck and *t*-*Z*-Ck mitigated the 75 and 150 mM NaCl stress impacts and significantly elevated SOD, CAT, APX, and GPX activities compared to the stressed 75 and 150 mM NaCl controls. Seed treatment with *t*-*Z*-Ck exceeded *c*-*Z*-Ck under both stress concentrations (75 and 150 mM NaCl), which exceeded the stressed control by 17.7% and 18.0% for SOD, 13.7% and 14.7% for CAT, 13.4% and 12.9% for APX, and 16.8% and 14.7% for GPX activity, respectively, under the stress conditions of 75 and 150 mM NaCl. The seed treatment was more effective under stress than under normal conditions (Figure 2).

### 3.7. Leaf Hormonal Content of Maize

**Figure 3:**
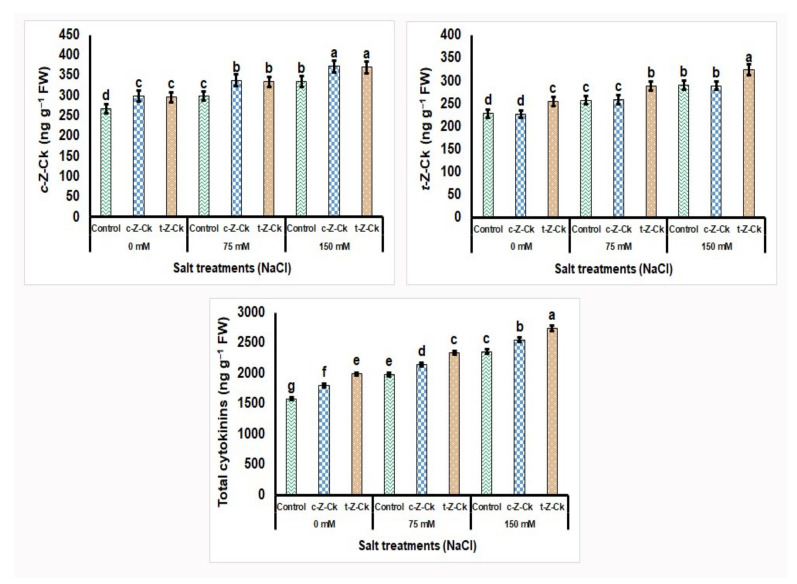
Seed soaking in 50-µM *c*-*Z*-Ck or 40-µM *t*-*Z*-Ck affected the leaf hormonal content (ng g^−1^ FW) of maize (Giza-168) plants exposed to salinity stress. Means with similar letters in each column are not significant at *p* ≤ 0.05, depending on Tukey’s test.

Seed pretreatment using 50 µM *c*-*Z*-Ck or 40 µM *t*-*Z*-Ck significantly increased the contents of *c*-*Z*-Ck, *t*-*Z*-Ck, and total cytokinins, with a minor fluctuation, compared to the control (Figure 3). Exposing maize plants to salt stress with 75 mM NaCl increased the leaf hormonal content of maize plants by 10.1%, 11.2%, and 20.2%, respectively, for *c*-*Z*-Ck, *t*-*Z*-Ck, and total cytokinins contents compared to the control. Further, exposing maize plants to salt stress with 150 mM NaCl further increased the leaf hormonal content by 27.6%, 29.3%, and 42.3%, respectively, for *c*-*Z*-Ck, *t*-*Z*-Ck, and total cytokinins contents compared with the control. However, seed treatment with *c*-*Z*-Ck and *t*-*Z*-Ck mitigated the 75 and 150 mM NaCl stress impacts and significantly elevated *c*-*Z*-Ck, *t*-*Z*-Ck, and total cytokinins contents compared to the stressed 75 and 150 mM NaCl controls, with a minor fluctuation compared to the salt-stressed control. Pretreatment with *t*-*Z*-Ck exceeded *c*-*Z*-Ck under both stress concentrations (75 and 150 mM NaCl), exceeding the stressed control by 10.7% and 10.2% for *t*-*Z*-Ck content and 15.4% and 13.9% for total cytokinins content, respectively, under the stress conditions of 75 and 150 mM NaCl. The seed treatment was more effective under stress than under normal conditions (Figure 3).

### 3.8. Leaf Content of Na^+^, Cl^−^ and K^+^, and K^+^/Na^+^ Ratio of Maize

Seed pretreatment using 50 µM *c*-*Z*-Ck or 40 µM *t*-*Z*-Ck did not affect the contents of leaf Na^+^, Cl^−^ and K^+^ ions and the ratio of leaf K^+^/Na^+^ in maize (Giza-168) compared to the control (Table 6). Exposing maize plants to salt stress with 75 mM NaCl increased leaf ion contents by 595.9% and 605.9%, respectively, for Na^+^ and Cl^−^ contents compared to the control. In contrast, it reduced K+ content by 22.11% and the K^+^/Na^+^ ratio by 88.8%. Further, exposing maize plants to salt stress with 150 mM NaCl further increased the leaf ion contents by 993.5% and 955.3%, respectively, for Na^+^ and Cl^−^ contents compared to the control. In contrast, it reduced the content of K^+^ and the ratio of K^+^/Na^+^ by 48.1 and 95.3, respectively. However, *c*-*Z*-Ck and *t*-*Z*-Ck pretreatment did not mitigate the 75 and 150 mM NaCl stress impacts and significantly reduced the leaf Na^+^ and Cl^−^ of maize plants compared to the stressed 75 and 150 mM NaCl control, while it increased the leaf K^+^ ion content and K^+^/Na^+^ ratio of maize compared to the stressed 75 and 150 mM NaCl control (Table 6).

### 3.9. Estimation of Yield and Yield Components

**Figure 4:**
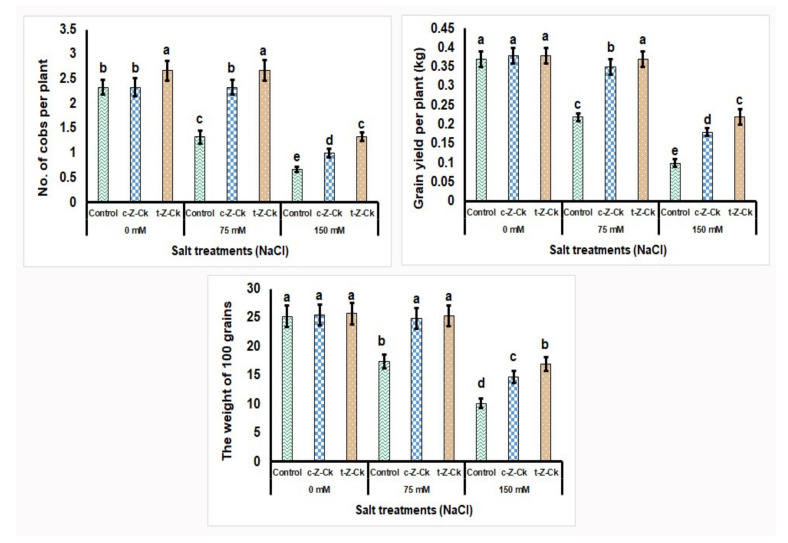
Seed soaking in 50 µM *c*-*Z*-Ck or 40 µM *t*-*Z*-Ck affected the maize yield and yield components (Giza-168) of plants exposed to salinity stress. Means with similar letters in each column are not significant at *p* ≤ 0.05, depending on Tukey’s test.

Seed pretreatment using 50 µM *c*-*Z*-Ck or 40 µM *t*-*Z*-Ck did not affect the number of cobs and grain yield per plant, as well as the weight of 100 grains of maize (Giza-168), with minor fluctuation (Figure 4). Exposing maize plants to salt stress with 75 mM NaCl decreased the number of cobs and grain yield per maize plant by 42.9% and 40.5%, respectively, as well as the weight of 100 grains by 31.0% compared to the control. Further, exposing maize plants to salt stress with 150 mM NaCl further decreased number of cobs and grain yield per plant by 71.2% and 73.0%, respectively, as well as the weight of 100 grains by 59.9% compared to the control. However, pretreatment with *c*-*Z*-Ck and *t*-*Z*-Ck mitigated the 75 and 150 mM NaCl stress impacts and significantly elevated plant growth and yield components compared to the stressed 75 and 150 mM NaCl control. Seed treatment with *t*-*Z*-Ck outperformed *c*-*Z*-Ck under both stress concentrations (75 and 150 mM NaCl), which exceeded the stressed control by 12.7% and 24.81% for the number of cobs per plant^−1^, 5.41% and 18.18% for grain yield per plant^−1^, and 2.0% and 13.02% for the 100 grain weight, respectively, under the stress conditions of 75 and 150 mM NaCl (Figure 4).

### 3.10. Relationship between Different Treatments and the Parameters Studied

**Figure 5:**
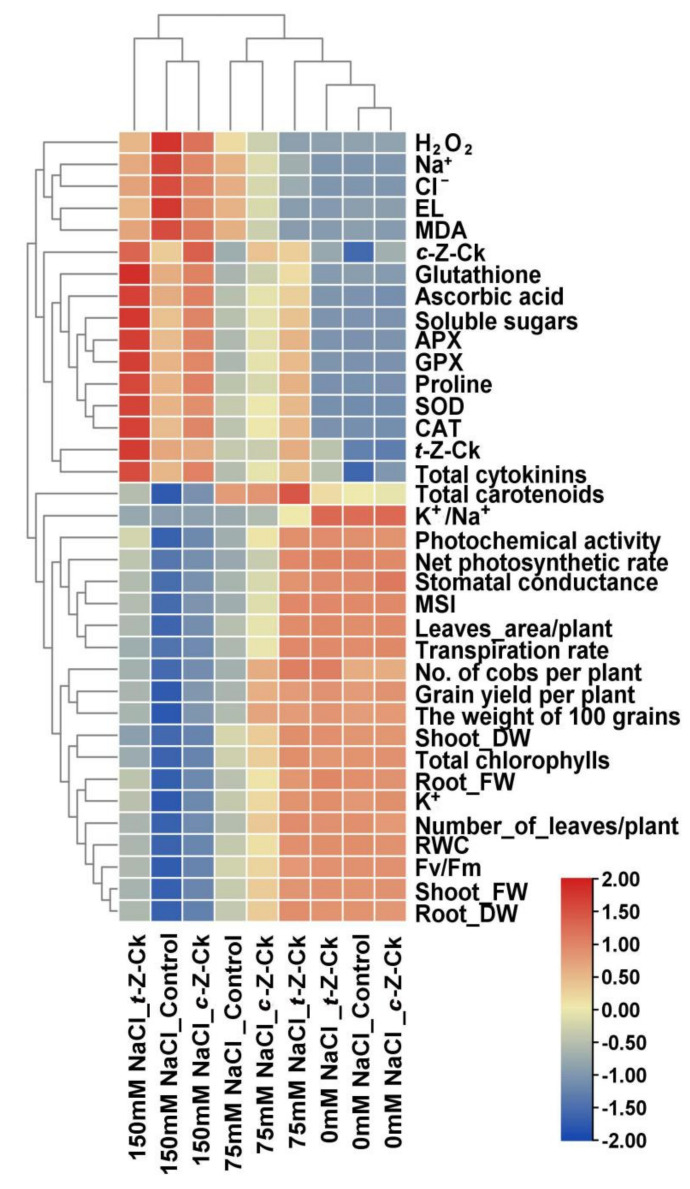
Heatmap graph showing the relationships and the hierarchical clustering analysis between the different treatments (control, *c*-*Z*-CK, and *t*-*Z*-CK) and the studied parameters of the maize plants grown under three levels of NaCl stress (0, 75, and 150 mM). SOD = superoxide dismutase, CAT = catalase, APX = ascorbate peroxidase, GPX = glutathione peroxidase, RWC = relative water content, MSI = membrane stability index, EL = ion leakage, DW = dry weight, FW = fresh weight, MDA = malondialdehyde, H_2_O_2_ = hydrogen peroxide. The colors represent the data variations among the treatments.

Figure 5 displays a heatmap that shows the relationship between the different treatments and the studied parameters. The hierarchical analysis divided the different treatments into two main groups (150 mM NaCl tratment and the treatments of 0 and 75 mM NaCl). This indicated that the 150 mM NaCl level resulted in a highly negative impact on the growth and physio-biochemical traits of the plants. Further, these two main groups were divided into four sub-main groups, which included 0 mM NaCl_*c*-*Z*-Ck, 0 mM NaCl_control, 0 mM NaCl_*t*-*Z*-Ck, and 75 mM NaCl_*t*-*Z*-Ck (in the first sub main group); 75 mM NaCl_*c*-*Z*-Ck and 75 mM NaCl_control (in the second sub-main group); 150 mM NaCl_*c*-*Z*-Ck and 150 mM NaCl_control (in the third sub-main group); and 150 mM NaCl_*t*-*Z*-Ck (in the fourth sub-main group). The overall results indicated that the *t*-*Z*-Ck treatment has a significant positive role in enhancing growth, yield components, physio-biochemical attributes, and the antioxidant system of maize plants. This positive finding indicated a mitigation of the adverse effects of NaCl stress by *t*-*Z*-Ck. Interestingly, the application of the 150 mM NaCl stress level increased the levels of H_2_O_2_, Na^+^, Cl^−^, EL, MDA, *c*-*Z*-Ck, glutathione, ascorbic acid, soluble sugars, APX, GPX, proline, SOD, CAT, *t*-*Z*-CK, and total CKs, while the K^+^/Na^+^ ratio, photochemical activity, net photosynthetic rate, stomatal conductance, MSI, total plant leaves area, transpiration rate, number of cobs and grain yield per plant, 100 grain weight, shoot DW, total chlorophylls, root FW, K^+^, number of leaves per plant, RWC, Fv/Fm, shoot FW, and root DW were decreased.

## 4. Discussion

Cytokinins (CKs) are phytohormones that promote cell division in plant root and shoot systems. They are classified into adenine and phenyl urea [51]. Phenyl urea CKs are not yet found in plants [52]. *Trans*- (*t-Z*-Ck) and *cis*-zeatins (*c*-*Z*-Ck), isopentenyladenine (IP), and dihydrozeatin riboside are the most common plant hormones [53,54]. To regulate the growth of plant shoots, *t*-*Z*-Ck is transferred from the roots to the shoot system [55]. Cytokinins participate in many physio-biochemical processes, including different cellular divisions and the senescence of leaves, thus regulating the ratio of shoot/root systems. As well, the long-distance transport of CKs is necessary for plants to respond efficiently to abiotic stresses such as salinity [56,57]. They play some regulatory roles (positive and negative) in reducing the harmful impacts of salinity. For example, the deficiency of CKs improves salinity tolerance and yield in many crops [58]. In plants, the primary enzyme (cytokinin oxidase; CKX) implicated in the metabolism of CKs can functionally suppress the concentrations of CKs. The hyposensitivity of many crops to salt stress is affected by CKX overexpression [58]. Excess CK production reduces ROS-scavenging enzyme transcript levels, resulting in an elevated production of ROS, which is linked to salt sensitivity [59]. Although there are adverse effects of CKs, other studies have demonstrated that CKs are beneficial for plant performance under salinity stress. In many crops, inhibiting CKX2 elevated CK levels and decreased yield penalties under saline stress conditions. AGO2 (the argonaute catalytic component RISC II) mainly contributes to enhancing salt stress tolerance by altering CK levels and increasing grain yield [58]. These contradictory studies warrant investigation of the handy use of CKs and propose tissue- and concentration-specific modes of action. Salt stress is frequently associated with lower concentrations of CKs in grain crops, and so the use of CKs increases grain yield [60].

The results showed that 50 µM *c*-*Z*-Ck or 40 µM *t*-*Z*-Ck were potent catalysts for growing maize plants against the studied salt stress. Following seed soaking, the CKs may translocate to the seed, allowing it to germinate quickly and strongly, resulting in a strong seedling that can effectively tolerate stress conditions. According to the findings of this study (Table 1, Table 2, Table 3, Table 4, Table 5, Table 6, Figure 2, Figure 3, Figure 4 and Figure 5), CKs mediated antioxidant system defenses and increased the K^+^/Na^+^ ratio to improve stress tolerance in maize plants. Under 75 or 150 mM NaCl stress, the decrease in the K^+^/Na^+^ ratio was due to the decreased uptake of K^+^ and the increased uptake of Na^+^ and Cl^−^ (Table 6). This finding was linked to reduced lipid peroxidation (malondialdehyde; MDA) and H_2_O_2_ levels (Table 5), which resulted in reduced plant growth and the inhibition of photosynthesis efficiency (Table 2, Table 3 and Table 4) and cellular metabolism (Table 5, Table 6, Figure 3, Figure 4 and Figure 5), with a loss of maize yield components (Figure 3 and Figure 5).

Due to the increase in energy requirements under stress, respiration increases and the metabolic processes of cells are disturbed, and thus the growth and productivity of the plant are restricted due to the decrease in the activities of meristems and cell expansions [61,62,63]. To deal with the undesirable consequences caused by the stress under the study, the maize plants upgraded their antioxidant systems, raising their antioxidant redox states, antioxidant enzymatic activities, and phytohormone levels (Table 1, Table 2, Table 3, Table 4, Table 5, Table 6, Figure 2, Figure 3, Figure 4 and Figure 5) [25,64,65,66,67,68]. Furthermore, soaking the seeds in CKs helped the plants’ antioxidant system to survive and maintain plant life under stress.

Under stress, in this study, maize plant growth and yield were better preserved due to stimuli that were further activated by seed soaking in both CKs (Table 1, Figure 3 and Figure 5). The improvements in the components of maize growth and production were attributed to increased efficiency of photosynthesis and uptake of nutrients, particularly K^+^ for Na^+^ ion antagonizing to decrease Na^+^ and Cl^−^ contents and to increase K^+^/Na^+^ ratio, all due to seed soaking in CKs (Table 2, Figure 3 and Figure 5) [17]. The exogenous application of CKs increased K^+^ ions and the K^+^/Na^+^ ratio in stressed plants, as confirmed in this study [69]. This positive ionic balance in stressed maize plants suggests that axial mechanisms were functioning in the stressed plant roots to minimize Na^+^ loading in the xylem. Furthermore, increasing the compartmentalization of Na^+^ was achieved, increasing the K^+^ ions in plant leaves [70]. This discovery resulted in an increased ratio of K^+^/Na^+^ in the cytosol, which serves as a critical indicator of salinity tolerance in plants. Furthermore, the enhancements in hormonal content (Figure 3 and Figure 5) and antioxidant defense system activity (Table 5, Figure 2 and Figure 5) caused by seed soaking in CKs increased the tolerance to salt stress (75 or 150 mM NaCl) in maize plants. The reduced MDA and H_2_O_2_ levels (oxidative stress markers; Table 5) under salt stress in plant tissues was due to seed soaking in CKs, assisting in increasing maize growth and yield components (Table 2, Figure 4 and Figure 5). Furthermore, CKs alleviated salt stress and improved photosynthetic machinery function (Table 2, Table 3 and Table 4) and cell metabolism [12,13]. Plant hormonal content increased under stress due to improved cell metabolism (Figure 3 and Figure 5). Specific plant responses to stress mediate the release of various plant hormones. CKs and their signaling ingredients primarily regulate plant defensive reactions based on the interaction of plants with stress [71]. Many plant species’ defensive responses to stress are modulated by CKs via defensive mechanisms, including the regulation of defensive genes and other hormones, such as ascorbic acid [72], which are CK-responsive [29]. This finding is supported by the findings about zeatins in this study (Figure 3 and Figure 5). These findings could be attributed to the fact that zeatins, as potential regulators of plant development and stress responses, have been discovered to have physiological functions throughout the plant [73]. In this study, seed soaking in *c*-*Z*-Ck or *t*-*Z*-Ck elevated the contents of *c*-*Z*-Ck, *t*-*Z*-Ck, and total CKs, and increased the plants’ tolerance to 75 or 150 mM NaCl (Table 1, Table 2, Table 3, Table 4, Table 5, Table 6, Figure 2, Figure 3 and Figure 4). Compared with *c*-*Z*-Ck, some reports have indicated that *t*-*Z*-Ck possesses generally higher activity. This superior performance of *t*-*Z*-Ck is attributed to its transport, conjugation, and degradation processes [73,74]. This result reflected the superior performance of *t*-*Z*-Ck treatment for all parameters tested in this study (Table 1, Table 2, Table 3, Table 4, Table 5, Table 6, Figure 2, Figure 3, Figure 4 and Figure 5), indicating greater induction of stress defense mechanisms and salt tolerance in maize plants. This positive finding is probably due to the physiological role of *t*-*Z*-Ck in conferring a greater increase in antioxidant accumulations under salt stress.

CKs primarily promote physiological responses by regulating gene expression [75,76]. However, little is known about the function of CKs at the molecular level under saline conditions. During oxidative stress, *Arabidopsis* CRF6 (CK responsive factor VI) suppresses cytokinin-linked genes [77]. A new, salt inducible TaCKX3 (CK oxidase/dehydrogenase) gene was discovered on chromosome 7B, silencing the TaCKX1 gene that elevated wheat yield [78]. CK treatment inhibited the expression of the high-affinity K^+^ transporter AtHKT1.1, which regulates xylem Na^+^ loading in Arabidopsis [79]. In addition, genes implicated in the breakdown of ROS are highly affected in the CK-deficient mutant ipt1,3,5,7 [59]. CKs aid in inducing the ERF-VI subfamily cytokinin response factors (CRFs), which positively regulate osmotic stress tolerance [80,81,82]. Otherwise, studies have discovered that excessive CK production harms plants by modulating stress-responsive gene expression. Under salt stress, the overexpression of CK biosynthetic gene AtIPT8 (adenosine phosphate–isopentenyl transferase VIII) prevents the emergence of the true leaf and growth of the primary roots. Further, it contributes to the excessive increase of ROS, which lowers the survival rate and chlorophylls contents, resulting in a reduced tolerance to saline conditions [59]. These multidirectional influences call for the practicability of CK use, implying that balanced levels of CKs are critical for plant adaptation to saline conditions. As well, the improved cell metabolism caused by CKs pretreatment activated the components of the antioxidant defensive system (Table 5, Figure 2 and Figure 5). This finding participated in scavenging excess ROS, preventing plasma membrane oxidation, and lowering MDA and H_2_O_2_ levels under saline conditions (Table 5). The physiological interactions between CKs and other distinct defense mechanisms mediated tolerance stimulated by other enhanced traits, such as proline, ascorbate, glutathione, antioxidative enzyme activity, and so on (Table 5). This finding could improve overall stress responses and plant tolerance to stress [8,68]. Incorporating different defensive mechanisms that are regulated by CKs identifies the efficacy in reducing the adverse effects of stress and modifies the physiological state of the limiting trade-off associated with the defense response. Pretreatment with CKs as pivotal bio-stimulators and growth promoters increased antioxidant and hormonal levels and suppressed ROS levels concomitantly with decreasing the peroxidation of lipids (MDA) and H_2_O_2_ level and increasing plant productivity (Table 2, Table 3, Table 4, Table 5, Table 6, Figure 2, Figure 3, Figure 4 and Figure 5).

According to this study’s findings, maize plant pretreated with CKs can survive better in stressed environments. These findings may be because CKs stimulated a significant increase in proline metabolism via the analysis of two pathways: anabolism and catabolism of P5CS and ProDH, respectively, which induce lower and higher P5CS and ProDH activities, respectively, to evenness proline levels in plant tissues [12,13,65]. CKs also effectively reduced H_2_O_2_ and MDA accumulations and membrane ion leakage (EL), thereby alleviating stress-induced oxidative damage (Table 5). It effectively contributed to the accumulation of osmoprotectant (soluble sugars and proline) levels to protect cells by maintaining a balance between the cytosol osmotic strength, the vacuole osmotic strength, and the osmotic strength of the external environment [83]. As unique biochemical stress signals, antioxidant enzymes are highly activated by CKs to alleviate the oxidative stress stimulated by salt stress. Therefore, maize plants pretreated with CKs had lower oxidative damage under stress after further activation of SOD, CAT, GPX, and APX (Figure 2 and Figure 5). Iqbal et al. [84] reported that exogenously used CKs increased plant antioxidant enzyme activities and suppressed ROS levels. As a consequence, the effects of salt stress are mitigated by minimizing Na^+^ and Cl^−^ uptake and maximizing K^+^ uptake. CKs (*c*-*Z*-Ck or *t*-*Z*-Ck) improved all components related to growth, yield, biochemistry, physiology, and the antioxidant defense system of the maize plants (Table 1, Table 2, Table 3, Table 4, Table 5, Table 6, Figure 2, Figure 3, Figure 4 and Figure 5). These findings could be due to CKs having high antioxidant activities and underlying mechanisms, including increased defensive antioxidants and minimized effects of oxidative stress and lipid peroxidation [84]. As a result, the use of CKs is a sustainable strategy. In some studies, plants of some crops are protected from certain stresses, including nutrient deficiency, salinity, and cadmium due to the plant’s defense system, which is rich in various antioxidants [12,13,85,86,87].

## 5. Conclusions

Cytokinins (CKs) are key regulators of plant growth and development. Recently, their participation in plant adaptations to salt stress and other stressors has been demonstrated. The most common types of CK are *trans*-zeatin (*t*-*Z*-Ck) and *cis*-zeatin (*c*-*Z*-Ck). It has been shown in several plant species, including Zea mays, that *t*-*Z*-Ck is a more biologically active CK. *Trans*-zeatin is more physiologically active than *cis*-zeatin in crop plants like maize. In addition, it has higher activity under stress conditions than *cis*-*Z*-Ck for transport, conjugation, and degradation processes, indicating a greater stimulation of stress defense mechanisms in maize plants. Compared with *c*-*Z*-Ck, *t*-*Z*-Ck induced greater tolerance to salt stress in maize plants, which is probably due to the physiological role of *t*-*Z*-Ck in conferring a greater increase in antioxidant accumulations under salt stress.

## Figures and Tables

**Table 1:** Seed soaking in 50 µM *c*-*Z*-Ck or 40 µM *t*-*Z*-Ck affected the growth and yield components of maize (Giza-168) plants exposed to salinity stress.

Salt Treatment (NaCl)	Zeatins Application	Shoot Fresh Weight (g)	Shoot Dry Weight (g)	Root Fresh Weight (g)	Root Dry Weight (g)	Number of Leaves Plant^−1^	Leaves Area (Dm^2^ Plant^−1^)
0 mM	Control	86.4 ± 8.0 a	38.9 ± 3.7 a	51.2 ± 4.8 a	20.5 ± 1.8 a	8.32 ± 0.76 a	34.8 ± 3.2 a
	*c*-*Z*-Ck	86.6 ± 8.3 a	39.0 ± 3.8 a	50.9 ± 4.6 a	20.3 ± 1.8 a	8.15 ± 0.74 a	35.1 ± 3.2 a
	*t*-*Z*-Ck	86.7 ± 7.9 a	39.5 ± 4.0 a	51.9 ± 4.8 a	20.5 ± 1.9 a	8.28 ± 0.73 a	35.2 ± 3.3 a
75 mM	Control	65.0 ± 6.7 c	30.8 ± 3.1 c	38.5 ± 3.2 c	15.3 ± 1.3 c	6.08 ± 0.55 c	24.4 ± 2.2 c
	*c*-*Z*-Ck	76.7 ± 7.2 b	35.0 ± 3.2 b	43.7 ± 4.5 b	18.2 ± 1.8 b	7.43 ± 0.72 b	27.7 ± 2.5 b
	*t*-*Z*-Ck	86.1 ± 8.4 a	39.6 ± 3.8 a	50.5 ± 4.8 a	20.7 ± 1.8 a	8.36 ± 0.83 a	34.9 ± 3.2 a
150 mM	Control	41.5 ± 4.3 f	20.2 ± 2.1 f	27.1 ± 2.9 e	10.0 ± 0.9 e	4.31 ± 0.34 e	16.1 ± 1.4 e
	*c*-*Z*-Ck	49.9 ± 4.7 e	22.8 ± 2.1 e	32.0 ± 3.1 d	11.6 ± 1.0 d	5.11 ± 0.40 d	19.9 ± 1.5 d
	*t*-*Z*-Ck	59.6 ± 6.3 d	25.4 ± 2.4 d	38.8 ± 3.3 c	14.5 ± 1.3 c	5.93 ± 0.52 c	23.7 ± 2.1 c
LSD at *p* ≤ 0.05		1.6	0.32	1.1	0.29	0.31	4.2

In each column, means (± SE) with similar letters indicate no significance at *p* ≤ 0.05, depending on Tukey’s test.

**Table 2:** Seed soaking in 50 µM *c*-*Z*-Ck or 40 µM *t*-*Z*-Ck affected the leaf pigments and photosynthesis efficiency of maize (Giza-168) plants exposed to salinity stress.

Salt Treatment (NaCl)	Zeatins Application	TChls	TCars	Fv/Fm	Photochemical Activity
		(mg g^−1^ FW)			
0 mM	Control	2.68 ± 0.04 a	0.86 ± 0.01 c	0.83 ± 0.01 a	45.2 ± 1.1 a
	*c*-*Z*-Ck	2.70 ± 0.05 a	0.85 ± 0.01 c	0.83 ± 0.01 a	44.9 ± 1.2 a
	*t*-*Z*-Ck	2.66 ± 0.04 a	0.88 ± 0.01 c	0.83 ± 0.02 a	45.5 ± 1.2 a
75 mM	Control	1.89 ± 0.03 c	0.97 ± 0.02 b	0.69 ± 0.01 c	32.4 ± 0.9 c
	*c*-*Z*-Ck	2.29 ± 0.04 b	0.98 ± 0.02 b	0.75 ± 0.01 b	38.8 ± 1.0 b
	*t*-*Z*-Ck	2.72 ± 0.05 a	1.07 ± 0.02 a	0.82 ± 0.01 a	45.3 ± 1.3 a
150 mM	Control	0.94 ± 0.02 f	0.60 ± 0.01 f	0.51 ± 0.00 e	24.7 ± 0.7 f
	*c*-*Z*-Ck	1.22 ± 0.03 e	0.70 ± 0.01 e	0.57 ± 0.01 d	28.7 ± 0.7 e
	*t*-*Z*-Ck	1.56 ± 0.03 d	0.78 ± 0.01 d	0.65 ± 0.01 c	36.2 ± 1.0 d
LSD at *p* ≤ 0.05		0.18	0.07	0.06	3.4

In each column, means (± SE) with similar letters indicate no significance at *p* ≤ 0.05, depending on Tukey’s test. Fv/Fm = photosynthetic efficiency, TChls = total chlorophylls, and TCars = total carotenoids.

**Table 3:** Seed soaking in 50 µM *c*-*Z*-Ck or 40 µM *t*-*Z*-Ck affected the leaf gas exchange of maize (Giza-168) plants exposed to salinity stress.

Salt Treatment (NaCl)	Zeatins Application	Net Photosynthetic Rate	Transpiration Rate	Stomatal Conductance
0 mM	Control	8.98 ± 0.16 a	7.07 ± 0.15 a	0.62 ± 0.02 a
	*c*-*Z*-Ck	8.91 ± 0.15 a	7.11 ± 0.14 a	0.64 ± 0.02 a
	*t*-*Z*-Ck	9.02 ± 0.16 a	7.10 ± 0.13 a	0.62 ± 0.02 a
75 mM	Control	6.12 ± 0.14 c	5.26 ± 0.10 c	0.44 ± 0.01 c
	*c*-*Z*-Ck	6.84 ± 0.12 b	5.92 ± 0.12 b	0.49 ± 0.02 b
	*t*-*Z*-Ck	8.94 ± 0.15 a	7.12 ± 0.15 a	0.61 ± 0.02 a
150 mM	Control	5.14 ± 0.11 d	4.22 ± 0.08 d	0.34 ± 0.01 e
	*c*-*Z*-Ck	5.61 ± 0.12 d	4.56 ± 0.09 d	0.39 ± 0.01 d
	*t*-*Z*-Ck	6.69 ± 0.13 b	5.08 ± 0.11 c	0.45 ± 0.02 c
LSD at *p* ≤ 0.05		0.56	0.50	0.04

In each column, means (± SE) with similar letters indicate no significance at *p* ≤ 0.05, depending on Tukey’s test.

**Table 4:** Seed soaking in 50 µM *c*-*Z*-Ck or 40 µM *t*-*Z*-Ck affected the relative water content (RWC), membrane stability index (MSI), and ion leakage (EL%) of maize (Giza-168) leaves exposed to salinity stress.

Salt Treatment (NaCl)	Zeatins Application	RWC (%)	MSI (%)	EL (%)
0 mM	Control	81.4 ± 5.3 a	77.8 ± 4.4 a	17.4 ± 2.2 e
	*c*-*Z*-Ck	80.9 ± 4.5 a	77.7 ± 4.6 a	17.3 ± 2.1 e
	*t*-*Z*-Ck	81.5 ± 5.1 a	78.0 ± 4.8 a	17.0 ± 2.2 e
75 mM	Control	62.2 ± 4.0 c	52.4 ± 3.5 c	27.6 ± 3.5 c
	*c*-*Z*-Ck	69.8 ± 4.6 b	61.3 ± 4.1 b	22.4 ± 3.2 d
	*t*-*Z*-Ck	81.3 ± 5.2 a	78.1 ± 4.7 a	17.2 ± 2.1 e
150 mM	Control	43.5 ± 3.1 e	40.1 ± 3.2 e	35.8 ± 4.1 a
	*c*-*Z*-Ck	49.8 ± 3.4 d	47.6 ± 3.8 d	30.2 ± 3.8 b
	*t*-*Z*-Ck	58.7 ± 4.3 c	55.2 ± 4.4 c	27.4 ± 3.2 c
LSD at *p* ≤ 0.05		6.2	5.8	2.1

In each column, means (± SE) with similar letters indicate no significance at *p* ≤ 0.05, depending on Tukey’s test.

**Table 5:** Seed soaking in 50 µM *c*-*Z*-Ck or 40 µM *t*-*Z*-Ck affected the lipid peroxidation, osmoprotectants, and antioxidants contents of maize (Giza-168) plants exposed to salinity stress.

Salt Treatment (NaCl)	Zeatins Application	MDA	H_2_O_2_	Proline (µmol g^−1^ DW)	Soluble Sugars (mg g^−^ DW)	Ascorbic Acid	Glutathione
		µmol g^−1^ FW				µmol g^−1^ FW	
0 mM	Control	22.8 ± 0.3 e	11.4 ± 0.2 f	71.2 ± 1.2 f	10.4 ± 0.2 f	1.62 ± 0.02 g	0.91 ± 0.01 g
	*c*-*Z*-Ck	22.4 ± 0.4 e	11.5 ± 0.2 f	71.4 ± 1.4 f	10.4 ± 0.2 f	1.59 ± 0.02 g	0.90 ± 0.01 g
	*t*-*Z*-Ck	22.4 ± 0.3 e	11.2 ± 0.2 f	71.5 ± 1.4 f	10.6 ± 0.3 f	1.64 ± 0.02 g	0.90 ± 0.01 g
75 mM	Control	34.2 ± 0.5 c	18.8 ± 0.3 d	114.8 ± 1.9 e	15.2 ± 0.4 e	1.97 ± 0.03 f	0.99 ± 0.01 f
	*c*-*Z*-Ck	27.4 ± 0.4 d	15.5 ± 0.2 e	131.2 ± 2.2 d	18.7 ± 0.4 d	2.24 ± 0.04 e	1.08 ± 0.02 e
	*t*-*Z*-Ck	22.5 ± 0.3 e	11.2 ± 0.2 f	184.6 ± 2.8 c	22.8 ± 0.4 c	2.46 ± 0.04 d	1.21 ± 0.02 d
150 mM	Control	41.6 ± 0.7 a	30.4 ± 0.4 a	182.8 ± 3.0 c	23.0 ± 0.5 c	2.68 ± 0.05 c	1.34 ± 0.02 c
	*c*-*Z*-Ck	38.1 ± 0.7 b	26.1 ± 0.4 b	216.5 ± 3.4 b	27.8 ± 0.5 b	2.94 ± 0.05 b	1.45 ± 0.02 b
	*t*-*Z*-Ck	35.1 ± 0.6 c	21.4 ± 0.3 c	254.2 ± 3.8 a	34.2 ± 0.6 a	3.32 ± 0.06 a	1.68 ± 0.03 a
LSD at *p* ≤ 0.05		2.2	1.4	10.4	1.8	0.21	0.09

In each column, means (± SE) with similar letters indicate no significance at *p* ≤ 0.05, depending on Tukey’s test.

**Table 6:** Seed soaking in 50 µM *c*-*Z*-Ck or 40 µM *t*-*Z*-Ck affected the contents of Na^+^, Cl^−^, and K^+^ and the ratio of K^+^/Na^+^ of maize (Giza-168) plants exposed to salinity stress.

Salt Treatment (NaCl)	Zeatins Application	Na^+^	Cl^−^	K^+^	K^+^/Na^+^
		mg g^−1^ DW			
0 mM	Control	1.23 ± 0.03 f	1.88 ± 0.05 f	2.08 ± 0.07 a	1.69 ± 0.04 a
	*c*-*Z*-Ck	1.24 ± 0.03 f	1.91 ± 0.06 f	2.11 ± 0.08 a	1.70 ± 0.04 a
	*t*-*Z*-Ck	1.23 ± 0.02 f	1.89 ± 0.06 f	2.12 ± 0.08 a	1.72 ± 0.04 a
75 mM	Control	8.56 ± 0.22 c	13.27 ± 0.40 c	1.62 ± 0.05 c	0.19 ± 0.00 d
	*c*-*Z*-Ck	5.21 ± 0.16 d	7.62 ± 0.21 d	1.84 ± 0.06 b	0.35 ± 0.01 c
	*t*-*Z*-Ck	2.67 ± 0.07 e	3.82 ± 0.17 e	2.10 ± 0.07 a	0.79 ± 0.02 b
150 mM	Control	13.45 ± 0.36 a	19.84 ± 0.58 a	1.08 ± 0.04 e	0.08 ± 0.00 f
	*c*-*Z*-Ck	10.57 ± 0.28 b	16.21 ± 0.46 b	1.31 ± 0.04 d	0.12 ± 0.00 e
	*t*-*Z*-Ck	8.92 ± 0.24 c	14.12 ± 0.42 c	1.58 ± 0.05 c	0.18 ± 0.00 d
LSD at *p* ≤ 0.05		1.12	1.38	0.19	0.04

In each column, means (± SE) with similar letters indicate no significance at *p* ≤ 0.05, depending on Tukey’s test.

## Data Availability

The data presented in this study are available upon request from the corresponding author.
